# Distributing epistemic and practical risks: a comparative study of communicating earthquake damages

**DOI:** 10.1007/s11229-022-03838-0

**Published:** 2022-08-22

**Authors:** Li-an Yu

**Affiliations:** Department of Philosophy, Bielefeld University, Bielefeld, Germany

**Keywords:** Science and values, Risks, Science communication, Openness to epistemic plurality, Social responsiveness

## Abstract

This paper argues that the value of openness to epistemic plurality and the value of social responsiveness are essential for epistemic agents such as scientists who are expected to carry out non-epistemic missions. My chief philosophical claim is that the two values should play a joint role in their communication about earthquake-related damages when their knowledge claims are advisory. That said, I try to defend a minimal normative account of science in the context of communication. I show that these epistemic agents when acting as communicators may encounter various epistemic and practical uncertainties in making their knowledge claims. Using four vignettes, I show that the value of openness to epistemic plurality and the value of social responsiveness may best serve their epistemic and practical purposes across different contexts by reducing their epistemic and practical risks associated with the knowledge claims they communicated. The former may reduce the risks of prematurely excluding epistemic alternatives and is conducive to two types of epistemic plurality; the latter is supposed to reduce the risks of making self-defeating advisory claims and harmful wishful speaking by minimizing the values in tension that can be embedded in the social roles the epistemic agents play.

## Epistemic and practical uncertainties and values in tension

A single earthquake with a magnitude of 7 on the Richter scale does not necessarily cause damages if it occurs in some uninhabited abyss. However, it would be another story if it happens in Tokyo, Beijing, or San Francisco as various, direct or indirect socioeconomic damages may be expected to occur in these populated areas (Radtke & Weller, 2021). In such cases, earthquakes become of general interest to scientists, policymakers, and society at large. This paper seeks to analyze and evaluate advisory knowledge claims about earthquake-related damages made by historical epistemic agents who were expected to carry out their respective non-epistemic missions, and relevant socioeconomic consequences resulting from their claims. I aim to provide epistemic agents of this kind, such as seismologists and civil engineers, as well as potentially wider audiences, such as public health experts, economists, and climate scientists, with some normative guidance in communicating their knowledge in the public space.

In order to defend my minimal normative account of science in the context of communication, I argue that the value of openness to epistemic plurality (OEP hereafter) and the value of social responsiveness (SR hereafter) should have been best suited for reducing their epistemic and practical risks when these epistemic agents “communicated” their knowledge for policy or action. In this paper, the term “risk” means uncertain harmful consequences. Risks can be reduced in the sense that relevant epistemically and practically harmful consequences would not result from inappropriate speech acts. Harms, however, could still result from other factors such as false understanding, ineffective action, and insufficient vigilance, resources, and time for preparation, which are not the objects of study in this paper.

In other words, my proposal is to tackle normative issues in epistemic agents’ speech acts potentially bringing about epistemically and socially harmful consequences. Moreover, my more ambitious goal is to add substance to the ethics of science communication, where communication norms such as openness and honesty are rejected to obtain their fundamental importance (John, 2018). In this paper, I use the term “communication” in a rather narrow sense, meaning speech acts or ways of speaking. In short, OEP encourages epistemic agents to avoid the premature exclusion of epistemic alternatives, which is furthermore conducive to two types of epistemic plurality (discussed in Sect. 3); For SR, I advance Kitcher’s account of the socially responsible scientist with an additional duty to prevent oneself from making self-defeating knowledge claims (discussed in Sect. 4).

My proposal to defend the minimal normative account of science in the context of communication comes up in the light of practical difficulties based on various epistemic and non-epistemic values in tension. Below I discuss three types of value-based uncertainty when epistemic agents have to (1) manage the information involved, (2) estimate losses, and (3) choose a timeframe of interest to focus on. They are three main channels where knowledge claims involved can result in conflicting advice. This typology aims to conceptually characterize the claims made by the historical epistemic agents in Sect. 2, and it prepares the ground for my analyses in Sects. 3 and 4. The value tensions in this typology are not exhaustive but enumerative. The listed tensions are used to show that values are not generally conducive to *reducing* epistemic and practical uncertainties because all of them could be valid evaluative benchmarks by themselves in specific contexts. These values in tension could, therefore, *sustain* epistemic plurality in scientific research as well as multiple socioeconomic and political demands on the use of the earthquake sciences. Moreover, this highlighted typology may cast doubt, again, on the conventional wisdom in the philosophy of science literature that values may help solve the problem of underdetermination and the problem of inductive risk (Brown, 2013). However, although my proposal may not directly resolve the value tensions, the two emphasized values about science communication should demonstrate their potential to reduce communication-induced, epistemically and socially harmful consequences.

### Managing information

The first value-based uncertainty appears as the epistemic agent needs to manage relevant information in forming advice. The two examples below show that values in tension may result in conflicting advice in managing information in order to achieve practical goals (Pielke, 2007; Sarewitz, 2004; Rayner, 2012).

For instance, the U.S. government sought to secure a permanent nuclear waste repository. Policymakers started with their policy to be *consistent* with the recommendation based on nuclear experts’ models reassuring the stability of the geological conditions of the Yucca Mountain nuclear waste repository (Shrader-Frechette, 2014). The reevaluations of the chosen location since the 1980s had been performed to justify the planned policy. The tension between the values of consistency and accuracy appeared between different expert groups as geological *accuracy* was pursued by drilling. These reevaluations by geologists turned out to reveal nuclear experts’ ignorance of its actual geological conditions, which were inconsistent with the models. Information about the seismic risk of the chosen location unsettled the originally planned policy, assuming its practical manageability and safety. The government’s goal of securing a permanent nuclear waste repository was then practically unachieved (ISSO, 2012). In this case, the conflicting pieces of information produced by the experts involved furthermore increased the government’s practical uncertainty.

The other example shows the tension between the values of accuracy and simplicity arising in mapping seismicity to minimize earthquake damages. The global mapping of seismicity produced by the international seismological community in the early 20th century largely helped narrow down the scientific focus on specific seismic areas on the Earth (Agnew, 2002; Bolt, 2003; Westermann, 2011). This achievement demanded *accurate* observations and records through seismological instrumentation and archival research, which was conducive to the development of mechanistic explanations of earthquakes and the improvement of earthquake-resistant designs. Nevertheless, endlessly accurate descriptions of past earthquakes did not exactly solve the problem of reducing earthquake damages. A proper solution was expected to have a sort of predictive power with *simple* patterns. To attempt a forecast (while some aimed at a prediction) of a catastrophic earthquake at a specific point in time, researchers had to actively reduce accuracy in establishing some simple relationships by focusing on chosen parameters such as that between main shocks and aftershocks (Omori’s law) and that between magnitude and occurrence (Gutenberg-Richter law). As the different demands of timeframes can be a limiting factor for these mentioned pursuits (Shaw, 2022), without decreasing accuracy, these modelers could not achieve any “predictive” goal (Cartwright, 1983; Hitchcock & Sober, 2004; Forsyth, 2011; Bokulich, 2013). However, “predicting” an earthquake, i.e., anticipating its magnitude on a specific spatiotemporal range in quantitative terms, still belongs to future science (Agnew, 2002; Oreskes, 2015; Hough, 2016; Stark & Freedman, 2016; Mulargia et al., 2017). In short, in order to approach reasonable advice on socioeconomic impacts of earthquakes with different demands of time, seismological research requires such values in tension as described.

### Estimating losses

The second value-based uncertainty appears as the epistemic agent makes an advisory claim in view of various socioeconomic losses, which should be understood as a more general consideration of inductive risk (Douglas, 2000). However, inductive methods such as statistical and probabilistic inferences in the classical toxicological case study provide few indications regarding a preferable default hypothesis to be tested, because the consequences of incorrectly accepting a false positive or a false negative in seismological research seem to be equally undesirable. Moreover, it appears to be difficult to agree on a “threshold” for acceptance in non-experimental settings as the dominant “risk assessment” of earthquakes in probabilistic terms is criticized to be epistemically inappropriate and socially harmful (Mulargia et al., 2017). The reason for such criticism is that quantitative expressions of uncertain harmful consequences often give misleading messages of epistemic certainty and practical manageability (discussed in Sect. 4).

In making a “predictive claim” about earthquakes, the epistemic agent, ideally, has to minimize socioeconomic losses by maximizing true positives and true negatives in her claims which remains practically unavailable (ISSO, 2012; Mulargia et al., 2017; Stark & Freedman, 2016). In doing so, she has to be cautious about harmful consequences from overestimating due to mistakenly accepted false positives, such as fear, long-term economic depression, or overinvestment in unaffordable earthquake-resistant designs. She also has to be cautious about harmful consequences from underestimating due to mistakenly accepted false negatives such as immediate casualties and damages resulting from insufficient preparation and lack of vigilance. To arrive at appropriate advice, weighing between these two types of errors in the light of various harmful consequences may thus reflect values in tension (de Melo-Martín & Intemann 2016).

### Choosing timeframes

The last value-based uncertainty appears as the epistemic agent must choose a timeframe for a catastrophic scenario in discussion. Such a choice may assume various values in tension. In addition, the epistemic imminence complicates such a choice because there is no reliable reason to exclude that a catastrophic earthquake will not occur in the immediate future (Hough, 2016; Oreskes, 2015). For instance, it may be possible to assume that a catastrophic earthquake will happen in a few years, a few decades, or even a few centuries. Scientific specifications for choosing such a timeframe require statistical approaches to historical earthquakes and seismic mechanisms. However, many regions with a catastrophic earthquake were not even mapped with high risk before the actual occurrence of an earthquake (Mulargia et al., 2017).

Think, for example, about the investment in the seawall prior to the Fukushima Daiichi nuclear disaster. The designers of the nuclear power plant had to decide on the height of the seawall as being key to protecting the reactors from tsunamis. Although they had recognized the possibility of an earthquake of this magnitude, a higher seawall, however, would demand higher construction costs, which could appear to be higher than necessary as the likelihood of a tsunami was very low. A catastrophic earthquake may have been perceived as occurring in a very distant future so its risk was mistakenly ignored. This underinvestment due to the chosen timeframe for a catastrophic scenario thus led to a seawall lower than necessary to protect the reactors.

On the contrary, similar considerations about choosing such a timeframe may influence the costs of earthquake-resistant designs. To increase the safety of a city, one might find it desirable to tighten building regulations so that increasing the costs of earthquake-resistant designs is required for improving long-term urban planning and resilience. Such a step, however, may result in increasing house prices on a short-term basis, which many people cannot afford. The latter might thus be unjustly excluded from the consideration of safer urban planning. It can be difficult to give general advice on improving urban earthquake resistance in view of the values in tension.

So far, Sect. 1 has characterized three types of value-based uncertainty that arise in the processes of making advisory claims: managing information, estimating losses, and choosing timeframes. The challenge is thus how individual epistemic agents, such as scientists who are expected to carry out non-epistemic missions, communicate their knowledge in ways that reduce their epistemic and practical risks in view of the values involved, which can result in conflicting advice. Note that such a challenge does not amount to finding ways to eliminate conflicting advice or values in tension or conflict.

The rest of this paper is organized as follows: Sect. 2 presents four vignettes to contextualize this typology of value-based uncertainty. The analyses of their associated epistemic and practical risks are presented in Sects. 3 and 4, which constitute my minimal normative account of science in the context of communication. Accordingly, Sect. 3 centers on OEP, and Sect. 4 on SR. The article concludes with Sect. 5.

## Four vignettes of advising in response to earthquake damages

Section 2 contextualizes the typology characterized in Sect. 1, using the advisory claims made by epistemic agents in response to earthquake damages. The four vignettes include the Church authorities in the 1755 Lisbon earthquake, the Yatoi architects in the 1891 Nobi earthquake, the commissioned scientists as well as “mass scientists” in the 1975 Haicheng and 1976 Tangshan earthquakes, and the commissioned scientists in the 2009 L’Aquila earthquake. These four vignettes provide a historically and geographically wide range of practical contexts, where epistemic agents made their advisory claims given sufficiently different non-epistemic missions.

The shared feature of these epistemic agents across contexts is that they were expected to carry out non-epistemic missions. This led them to make specific advisory claims about earthquake damages while committing to particular values. Their knowledge claims, however, resulted in social harms in relevant contexts. Thus, their credibility as epistemic agents was challenged (discussed in Sect. 4).

I suggest that these epistemic agents should have committed themselves to the minimal set of communication norms, that is, OEP and SR, in public, to reduce their communication-induced risks to science and society.

### The wrath of God, the ruin of churches, and the 1755 Lisbon earthquake

The first vignette is about the church authorities’ false reassurances regarding earthquake damages. During the 18th century, natural philosophy and natural history, and, more importantly, the church authorities still dominated the epistemic authority about nature in society (Rudwick, 2014). The common explanation of earthquakes was that they resulted from the wrath of God. In contrast, Kant’s reflection after the 1755 Lisbon earthquake and the development of his naturalistic account marked an interesting move in understanding such events (Reinhardt & Oldroyd, 1982, 1983).

Along with Buffon’s idea, Kant thought that these earthquakes could be explained with purely mechanistic and chemical explanations such as inflammable materials exploding in underground cavities (which is wrong in today’s seismology). Here, Kant took up the traditional task of naturalists collecting bits and pieces of nature with *accuracy* and explained the existence of earthquakes in a *simplistic* account without appealing to God. As for his practical purposes, based on his scientific account, Kant advised his readers to avoid earthquake damages by not living around underground cavities.

On the contrary, the situation after the Lisbon earthquake became painful for other intellectuals who favored deistic philosophy such as Voltaire because obviously “there is evil in the world” (Reinhardt & Oldroyd, 1983). They found that the cruel nature could not at all justify the benevolence of God. As this ground-shaking event occurred, unfortunately, on All Saints Day in 1755, numerous pious priests, nuns, and believers were all buried in the ruins of churches, while many of the impious or disbelievers survived (Reinhardt & Oldroyd, 1983; Musson, 2012). The result was not the one the church authorities had propagated: only disbelievers were to be punished by God. The Lisbon earthquake revealed that pious believers had apparently been punished by God for no reason. The epistemic authority of the churches was thus heavily challenged when people saw the cruelty of nature created by an omnibenevolent God. For instance, Voltaire, since then, relentlessly satirized and frequently referred to this catastrophic event as revealing the depravity of churches.

### Yatoi architects and the Meiji architectural programs before the 1891 Nobi earthquake

The second vignette is about the misleading advice of yatoi architects on improving the urban earthquake resistance. Employed by the newly organized Meiji government in Japan, the first generation of European intellectuals, including scientists and engineers, were called yatoi (雇), meaning “the employed.” Their missions were straightforwardly connected to the Japanese national policy of Westernization. Here, we focus on two of them from Britain who were responsible for various government-sponsored civil engineering and architectural projects, and later transformed the development of modern seismology.

The yatoi were hired by the government and worked at the newly founded College of Technology (工部大学校, today’s Faculty of Engineering at the University of Tokyo) since the 1880s. One of their most significant missions was to modernize Japanese buildings to build up a stronger empire of Japan (Clancey, 2006).

Japanese buildings of this period were characterized by the practices of traditional craftsmanship called daiku (大工), whose work could neither be identified with engineering nor architecture in the eyes of the British yatoi. For example, based on the Euclidean geometry, architects then often criticized the traditional heavy roof as an “irrational and redundant” design^1^ for a building in an earthquake nation. It should have been removed as soon as possible. The yatoi could offer an alternative based on a simpler model. Most of the yatoi were stunned by the fact that the main body of Japanese construction was made of wood, which was characterized as being “frail” and “temporary.” They wondered how buildings made of such materials could resist imminent catastrophic earthquakes. The call for urgent transformation of Japanese construction occurred hereafter.

By contrast, the mid-19th -century British architecture was characterized by geometrical and physical principles and “robust” and “permanent” materials: architectural design, brick, and stone. Along with this tradition, the founder of Japanese modern architecture and professor Josiah Conder (1852–1920) argued that it was essential to transform wooden Japanese construction based on the British model. He believed that this should be the essential move to modernize Japan and manage catastrophic earthquakes as well as urban fires. This epistemic commitment led Conder to ignore the documents that went against his theory, such as that by the designer Christopher Dresser (1834–1904) on thousand-year-old Buddhist pagodas. The known oldest one, Horyuji (法隆寺) in Nara, was built in the 7th century. These ancient buildings had been entirely made of “frail and temporary” materials and maintained by “irrational and redundant” practices of daiku.

As an intellectual opponent to Conder and a co-founder of the Seismological Society of Japan (日本地震学会) in 1880 (Clancey, 2006; Davison, 1937), the British professor of geology John Milne (1850–1913) cast serious doubt on the applicability of Conder’s project of bringing British architecture to Japan. This was due to his own experience of recording the moderate Tokyo-Yokohama earthquake in 1880 in light of his European seismology. This seismology then was developed by the Irish civil engineer Robert Mallet in recording earthquakes in Europe and South America. His method was to study the patterns of cracks imprinted on the shaken building structures made of stone and bricks. With these patterns based on observation Mallet could retrodict epicenters and depths of earthquakes on mechanics (Mallet, 1846; Gillin, 2020).

The difficulty that faced Milne’s research after the Tokyo-Yokohama earthquake was that Mallet’s method did not apply to wooden buildings at all. He had a difficult time recording the patterns of cracks or serious damage on Japanese wooden structures. Flexibility, rather than the mentioned frailness and temporality, characterized the Japanese constructions hit by this earthquake. Eventually, he found a proper “seismograph,” which was composed of a bunch of European buildings around Yokohama. However, it remained a problem for him to locate the epicenter by tracing the effects of the earthquake on the wooden structures beyond the areas without clustered European buildings.

On the same occasion, the epistemic tension between the architect and the geologist loomed large in an interesting way in their scientific interpretations of hundreds of ruined European-style buildings of the modern Ginza, which had been commissioned by the Meiji government in the 1870s. Until 1880, the modern Ginza was the model Japanese city completely composed of European-style buildings. It demonstrated a template for a nationwide modernization project of Japanese buildings to follow. The Tokyo-Yokohama earthquake in 1880 did not change Conder’s belief in modernizing Japan based on the British model. He could argue that the ruins of the model city were not so much about the British architectural design. Rather, it was due to the inability of the Japanese construction workers who could not follow the architectural plans and aptly use stone and bricks.

By contrast, Milne saw these systematically built columns of European buildings as an array of seismometers because the ruins did demonstrate some specific patterns such as the direction of the collapsed chimneys, which later became a research focus of his famous student Omori Fusakichi (大森 房吉). In addition, this experience led Milne to gather engineers and physicists at the College of Technology who were also interested in seismicity and advancing its instrumentation. This seismological community formed a new approach that was remarkably distinct from Mallet’s observational seismology. With the seismographs, Milne and his colleagues and students opened up instrument-based seismology, going beyond the previous limitations to recording simple patterns only on building structures. They rearranged seismology under the brand of geophysics, which should be studied in terms of physics, rather than solving problems of engineering. In Milne’s first seismology textbook published in 1886, he praised the flexibility of Japanese wooden construction and criticized the rigidity of Victorian architecture using masonry and bricks. He supposed the problem of the upending of European-style buildings was due to the wrong earthquake-resistant design because his architect colleagues did not understand the mechanics of earthquakes.

This intellectual conflict between Conder and Milne and their peers lasted until 1891 when the catastrophic Nobi earthquake occurred unexpectedly. The original Meiji architectural programs with the model Ginza terminated, and the ruins of the European-style buildings again attracted attention on the disadvantages of over-Westernized Japanese buildings. The government, intellectuals, and society started a reevaluation of the traditional Japanese wooden constructions. To build up a long-term seismic-resistant Japanese Empire, this apparently robust and permanent British architecture would not work on a short-term basis.

### Chinese mass seismologists and earthquake prediction in the 1970s

The third vignette is about commissioned scientists’ mistaken beliefs and strategies in earthquake prediction. The Cold War politics shaped seismology and disaster policy in Maoist China in dramatic fashion, in particular, the science of predicting earthquakes by learning from the people for the people. This form of cooperation between science and policy was characterized as being in contrast to Weberian, technocratic, value-free science.

The Communist party’s enthusiasm for predicting earthquakes appeared during the Cultural Revolution (1966–1976), which was an unusual period with more than ten large earthquakes killing a quarter of a million people in China Proper (Fan, 2012). As a result, the 1975 Haicheng earthquake and the 1976 Tangshan earthquake within a very short time, by luck, tested this enthusiasm for earthquake prediction and dashed it.

Traditionally, a large earthquake in China Proper would be rendered as a sign of political turmoil or an end to an emperor or dynasty (Agnew, 2002; Fan, 2012). This epistemic-political system assumes that *Tian* (the Heavens or nature) brings about a catastrophe because the emperor’s virtues are not strong enough to justify the continuation of his reign. The Communist party could feel this pressure when a large earthquake occurred. The solution the leaders came up with was to actively educate people that this belief was merely superstitious, and that, more importantly, earthquakes “could be predicted” in the spirit of the Engels-Maoist philosophy.

Mao believed that the goal of science is to “conquer nature, to free oneself from nature” (人定勝天), which is parallel to Engels’ “labor creates humanity.” That was the optimism of human capacity to intervene in the course of nature. For this, Mao emphasized the utilitarian value of science while dismissing the value of theoretical science such as Einstein’s theory of relativity. As long as earthquakes could be predicted, their socioeconomic impacts could be prevented. Seismological research was in this sense of utilitarian value and supported by the Communist government. The Chinese geologist Li Siguan (李四光, 1889–1971) and geophysicist Weng Wenbo (翁文波, 1912–1994), who endorsed this political commitment, helped initiate and implement the projects of predicting earthquakes during the time the Chinese scientific community became relatively isolated from the West since the beginning of the Cold War, and from the Soviet Union since the late 1950s.

During this period of isolation, Chinese seismology adopted an approach that was distinct from other countries (Fan, 2012). First, the Chinese scientists mapped the earthquakes in the historical records of thousands of years, which were lacking in their foreign counterparts. This helped in locating the places with a higher likelihood of earthquake occurrences and linked various precursory phenomena with large earthquakes. Second, instead of focusing on monitoring seismic activities with sensitive instruments, the Chinese scientists first focused on macroscopic phenomena that laypeople could observe, say, abnormal animal behavior. The scientists believed that these phenomena could be used to accurately predict earthquakes on a short-term basis. This was at the same time deemed unlikely by the international scientific community. Third, while lacking sufficient seismological stations and trained staff in the vast land, laypeople were advised to learn and do science by themselves to contribute to this nationwide defense policy. In other words, there was a demand from above to blur the distinction between scientific experts and laypeople for the epistemic and political goal of predicting earthquakes.

The government officials and teachers at all levels were enthusiastic about teaching people and students the science of predicting earthquakes by paying attention to the “precursory phenomena” of changes in biological behavior, water and sea levels, tides, ground deformation, climate, and weather as well as earthquake sounds and light. This required assistance from specialists in a wide range of scientific disciplines besides geophysics (Fan, 2012, 2017, 2018). According to this view, earthquakes were seen as a collection of experiences of complex changes in the environment rather than single geophysical events.

These efforts led to a successful evacuation in 1975. Until today, this is the only recognized case of a successful earthquake evacuation policy based on “earthquake prediction,” which had only a shaky scientific basis (Fan, 2012, 2017, 2018; Musson, 2012; Jordan et al., 2011; Hough, 2016). A couple of weeks before the Haicheng earthquake, there were reported cases of strange behavior of snakes crawling from their hibernation holes in the freezing-cold winter. A large portion of the population was evacuated a couple of hours before the main shock. People who had not been willing to follow the Party’s lead were blamed for not believing in the Party and thus were considered as having deserved their losses. Until the summer of next year, the Tangshan earthquake came without sufficient clues and time for an evacuation measure and caused hundreds of thousands of victims. The hope of predicting earthquakes for disaster policymaking was thus relentlessly dashed shortly after an ephemeral glory over “conquering nature.”

### Regretful scientist-officials after the 2009 L’Aquila earthquake

The last vignette is about commissioned scientists’ false reassurances of no danger. The lawsuits against the scientists after the L’Aquila earthquake constitute a nice example to rethink how scientists should communicate their knowledge under great uncertainty. Relevant debates on the relationships between scientific expertise and policymaking, and between the roles of scientists and policymakers remain until today (Mitchell, 2004; Pievani, 2012; Peters, 2021; Feldbacher-Escamilla, 2019).

In the trial in 2012, nearly three years after the L’Aquila earthquake, six scientist-officials in the governmental commission were sentenced to jail for failing to provide adequate information about the situation of the earthquake, which led to 309 deaths among other losses (Oreskes, 2015). The accusation was not the failure of predicting the earthquake itself, but how their advice misled the public (Feldbacher-Escamilla, 2019).

Regarding a series of swarms that happened in early 2009 before the mainshock, these scientist-officials claimed before the public that there was no evidence that such swarms can predict a large earthquake. They correctly indicated that any prediction of this kind was scientifically unwarranted. By contrast, a predictive claim had been made in the meantime by a retired technician who had worked in Gran Sasso National Laboratory. The technician of astrophysics Giampaolo Giuliani, who had long observed changes in the underground radon concentration, concluded his study without peer review along with the mentioned swarms and alarmed the public about a coming earthquake. Before long, Giuliani was officially forbidden to make claims of this kind in public.

Among the scientist-officials, the professor of volcanology Franco Barberi and the professor of hydraulics and then vice-director of the Department of Civil Protection Bernardo De Bernardinis openly criticized Giuliani’s predictive claim on television, and further reassured the public that the series of swarms would pose “no danger.” De Bernardinis even claimed that these swarms helped lessen the seismic potential by dissipating the accumulated seismic energy in this region.

Unfortunately, their reassurances turned out to be wrong. The mainshock came with serious damages. Their advisory and scientific claims had thus been false. The earthquake further disturbed the credibility of the scientist-officials who had not denied the potential falsity of their hypothesis and misled the public to believe that there would be no danger. To some degree, these scientist-officials even self-defeated their own claim that any prediction of this kind was scientifically unwarranted. Their claim of “no danger” should have been seen as a predictive claim about these earthquake damages as it suggested a probable development of the status quo (discussed in Sect. 4).

At least, in this case, the situation could have been better if the alarm by Giuliani had not been officially forbidden in public (discussed in Sect. 3). Without the false reassurances made by the scientist-officials, some deaths could have been avoided even if the victims merely followed their folk seismology and earthquake mitigation measures they inherited from their ancestors (Oreskes, 2015).

Table 1 provides a summary of the four vignettes and makes it easier for the reader to compare claims made by the above epistemic agents and their consequences. In the subsequent two sections, I use a quasi-inductive, counterfactual argument^2^ for each emphasized value across contexts. If OEP and SR were not absent in the processes of making advisory claims in the four vignettes, epistemic and practical risks resulting from communicating knowledge for action in the relevant contexts could have been reduced.

**Table 1:** Comparison of the four vignettes

Case	Advisory claims in question	Epistemic alternatives	Comparison of epistemic consequences	Comparison of non-epistemic consequences
Lisbon	The church authorities’ claim that only the impious and disbelievers will be punished by God	Kant’s mechanistic and chemical explanations	The church authorities’ claim that only the impious and disbelievers will be punished by God’s wrath failed. Kant’s attempt to bridge the divide between natural history and natural philosophy was generally correct	The credibility of the epistemic authority of the churches was challenged. Kant’s account directed attention to geological conditions, rather than God when aiming at reducing the harm from earthquakes
Nobi	The architect’s claim that British architecture is seismically resistant, with which traditional Japanese construction should be replaced	The seismologist’s claim that traditional Japanese construction in facing earthquakes is more seismically resistant than British architecture	The architect’s overconfidence in his theory was later challenged by a real earthquake. The seismologist noticed that the established seismology did not work in the Japanese context and tried to develop a new approach	The nationwide Westernization regarding buildings based on the British model was turned down when the difference in local damages between the traditional and the British buildings became obvious after a few earthquakes. The instrument-based seismological community launched
Haicheng & Tangshan	The government’s and scientists’ claim that earthquakes can be predicted on macroscopic precursory phenomena that laypeople can observe	The international scientific community’s claim that earthquake prediction is now highly unreliable	The prediction of the Haicheng earthquake succeeded. The quality of data collection from laypeople was unreliable in contrast to instrument-based seismology by the international scientific community	An evacuation policy based on “earthquake prediction” was successfully implemented once but not a second time, and led to a decrease in the social vigilance regarding earthquakes without sufficient precursory phenomena. The credibility of the government and scientists’ ideal of earthquake prediction was challenged. The costs of many false alarms were not calculated.
L’Aquila	The scientist-officials’ claim that the swarms could dissipate seismic energy and posed no danger	The retired technician’s claim that the swarms might indicate a coming earthquake. The folk seismology advising people to stay outdoors when frequent trembles appear	The scientist-officials’ claim was falsified by a real earthquake. Though scientifically unreliable, the heretical claim without peer-review and folk seismology in indicating the possibility of a coming earthquake could be harm-reducing	The credibility of the scientist-officials was challenged. Their reassurances misled the citizens’ risk aversion behavior and an indirect reason for their deaths

## The value of openness to epistemic plurality

In this paper, OEP is characterized as a value of communicating knowledge claims. It encourages epistemic agents to make a normative commitment by avoiding the premature exclusion of epistemic alternatives. It is, moreover, conducive to two types of epistemic plurality, which are of epistemic value and positively contribute to improving advisory claims.

Why did some historical epistemic alternatives appear to be eventually more successful? The reason was normally that they survived empirical scrutiny, while their competitors did not. If one is to unfairly stifle such alternatives “prematurely,” that means one gives a false verdict of them before such empirical scrutiny. Comparing the above four vignettes, I show that OEP should be, firstly, a communication norm for avoiding such a verdict so that some epistemic and practical possibilities would not be prematurely killed. And then it is conducive to enlarging a comprehensive knowledge basis for dealing with earthquake damages. Without this commitment, individual epistemic agents run the risk of distancing themselves from reaching these goals.

As can be seen, individual epistemic agents could make their advisory claims by committing themselves to particular values, which could be, to various degrees, in tension with other value commitments in relevant contexts shown in Sect. 1. The false reassurance of church authorities was *consistent* with their belief in such events as being God’s punishment of disbelievers. The yatoi architects gave their advice on transforming Japanese buildings without critically examining their mistaken assumption that British architecture should be directly adopted in Japan although their advice was *consistent* with their professional training in Britain. And the Italian commissioned scientists’ *simplistic* reassurances of no danger gave misleading advice to the public although scientists had long known that the real situation could be more complicated. Epistemic agents in these three cases exhibit epistemic resistance to considering more information. On the contrary, the Chinese commissioned scientists appeared to switch on all channels of information. They advised “mass scientists” to look for a very wide range of phenomena with *accuracy* to increase relevant information for predicting earthquakes.

The advisory claims, however, turned out to be epistemically and practically harmful because the epistemic agents were *not open to epistemic plurality* when communicating their advisory claims about earthquake-related damages to their audiences. Eventually, the exclusive advisory claims made by the church authorities, yatoi architects, and Italian commissioned scientists resulted in understating the earthquake damages they could experience by implicitly excluding short-termed damages. In contrast, the strategies of identifying precursory phenomena proposed by the Chinese commissioned scientists led to many false warnings, while the missed catastrophic one was seriously underestimated (discussed below). They gave a false impression that such damages could be managed on all timeframes of interest simply by looking at “mass observation” of all kinds of precursory phenomena.

Table 1 provides a comparison. The epistemic agents making these advisory claims in question were generally expected to carry out various non-epistemic missions, responding to potential earthquake-related damages, in particular. However, they failed this misplaced expectation while their advisory claims did not survive empirical scrutiny.

Their non-epistemic missions could have been better achieved if, first of all, their advisory claims had not been made to their audiences and they had not prematurely excluded alternative knowledge claims. Worse, dismissing the epistemic alternatives would create blind spots and mistakenly lead their audiences not to consider other options for action that could have brought about mitigating effects on the socioeconomic impacts of the earthquakes. In hindsight, their situations could have become better if OEP were appreciated by those making advisory claims. OEP, as a shared value commitment, sheds light on blind spots that individual epistemic agents could create so that a more comprehensive knowledge basis could be established for producing more options for action.

Here one might start feeling worried that the shortcomings of “epistemic plurality,” such as inappropriate dissent, will be included accordingly as one can see endless climate denialism in American society (Oreskes, 2004; Oreskes & Conway, 2010; de Melo-Martín & Intemann 2014; Leuschner, 2018; Kourany & Carrier, 2020). Stressing too much epistemic plurality in deliberating advice may delay necessary urgent action or sustain inaction by including conflicting knowledge.

However, “epistemic plurality” is not the value I seek to argue for in this paper. I do not suggest that individual epistemic agents should commit themselves to closely examining their opponents’ claims before coming up with their advice. Such examination takes time, energy, and resources which can be well in tension with the demand for urgent advice (Shaw, 2022). Instead, I argue that the value of “openness to epistemic plurality” as a communication norm is essential for these epistemic agents to “avoid the premature exclusion of epistemic alternatives.” In our four vignettes, this “avoidance of exclusion” itself would have already had a mitigating effect on the harmful consequences resulting from their mistaken advisory claims. For instance, if the warning from Giuliani had not been banned by the civil protection committee from the public fora, it could have kept members of society vigilant so they could have somehow avoided damages by autonomously considering other possible scenarios and taking their own protective actions. But that does not suggest the committee should have included whatever Giuliani said in their official announcement, including his empirically unscrutinized knowledge claims. They should just have avoided making false reassurances and creating blind spots that mistakenly narrowed down the public attention. This distinction between “exclusion” and “inclusion” is important because my emphasis on “avoidance of premature exclusion” aims at reducing the potential harms resulting from their advisory claims, instead of aiming at increasing inconsistency in their advice. Thus far, my characterization of OEP may be more relevant to time-sensitive actions such as communicating advisory claims of imminent earthquake damages.

In the four vignettes, the presented unforced *alternative plurality* of knowledge claims at a point should not have been actively excluded, as the epistemic alternatives could have constituted a harm-reducing factor for the mistaken advice. For instance, Table 1 shows that the insufficient vigilance regarding the Tangshan earthquake could have resulted from the commitment to observing precursory phenomena, but the international seismological community did not assume that all great earthquakes would come with such phenomena. Another example is yatoi architects’ dismissal of the earthquake resilience of Japanese daiku’s works, which was exactly the reason why cracks were not recorded on the traditional buildings. My suggested commitment to OEP seems to be a relatively acceptable solution when the time for critically examining opponents’ knowledge claims is limited and urgent advice is expected.

However, the time for examining opponents’ knowledge claims is not necessarily always limited or insufficient. Alternative plurality on the community level as a result of committing to OEP can be empirically scrutinized on a long-term basis. Therefore, OEP can be compatible with and conducive to increasing epistemic plurality in science for establishing its objectivity (Longino, 1990; Lacey 2005) by incorporating previously missing empirical scrutiny, which usually requires much time. The commitment to OEP does not exclude the possibility that epistemic agents can at some point reasonably exclude some alternatives, openly criticize inappropriate dissent, and avoid malicious epistemic plurality like climate denialism or vaccine hesitancy. If the epistemic community could constantly compare various knowledge claims, they are positioned to better understand their matters of concern. The reason is that the reliability of knowledge claims can be buttressed when the community sustains its ability to retain reliable knowledge claims and sift out unreliable ones through experience and interaction with nature. As the relevant knowledge basis increases its reliability, epistemic agents may know which advisory claims are safe to make and which are not. Thus, epistemic agents who commit themselves to OEP in communicating their knowledge can still benefit from *alternative plurality* and improve their advisory claims.

The other kind of epistemic plurality becomes clearer when epistemic agents appreciate some appropriate division of cognitive labor throughout the development of knowledge (Bokulich, 2013). It can be seen that the alternative knowledge claims in Table 1 could outperform the dominant views in their contexts. Even the alternative in the fourth vignette might seem controversial or potentially false, but the radon hypothesis is yet to be rejected conclusively. This gives epistemic agents an additional reason to consider committing to OEP not only when communicating their knowledge in public but also within their epistemic community. If these epistemic alternatives were unfairly dismissed and failed to be communicated within the epistemic community in the long run, they could run the risk of failing to develop a correct understanding of nature. For instance, one might still hold the misplaced confidence in the earthquake resistance of 19th -century Victorian architecture or the predictability of earthquakes. And such dismissal or failed communication of epistemic alternatives should be undesirable for improving advisory claims. Distributing the risk of misunderstanding nature by dividing a family of research questions into various epistemic alternatives has been a good strategy for approaching successful science on the community level (Wray, 2011; Bokulich, 2013). Additionally, the commitment to OEP encourages epistemic agents to avoid the premature exclusion of their opponents’ claims.

As a result, *additive plurality* is the plurality of these historical epistemic alternatives that add up to our successful understanding of nature. They could result from various disciplines, research programs, traditions, paradigms, communities, and so on. The wide range of disciplines about earthquake damages, such as the listed natural history, architecture, civil engineering, urban planning, geology, and seismology, constitute a representative case of the making of our contemporary understanding of seismic risk assessment. As the additive plurality of those successful knowledge claims adds up to a comprehensive knowledge basis, the failures of the knowledge claims demarcate where the limits of science may be. This complementary feature should be generally desirable for demarcating reasonable scientific advisory claims from unreasonable ones.

In sum, to avoid prematurely giving a false verdict of advisory claims and mistakenly rejecting knowledge claims or options for action, I suggest that epistemic agents should commit themselves to the value of openness to epistemic plurality which avoids epistemic and practical risks resulting from communicating their knowledge. This value should be conducive to and compatible with two types of epistemic plurality on the community level: alternative plurality and additive plurality. The former reduces the risk of being wrong about nature on a short-term basis as epistemic divides appear. The latter enriches the knowledge basis for being right about nature in the long run as various disciplines develop and interact with one another to increase their reliability and credibility. Both of them are supposed to positively contribute to deliberating reasonable scientific advice.

## The value of social responsiveness

Social responsiveness may mean different things for different people as one might think of the difference between what a socially responsible scientist and a socially responsible policymaker would do. The difference relies much on what society expects of these social roles which can change much across contexts. However, this fact does not mean that it is not possible to characterize respective social responsiveness more generally. For instance, given that political systems and social value commitments can vary strongly in different cultures, policymakers should still actively consider a sufficiently wide range of social values in their contexts to deliberate on policy consequences informed by science. This could prevent them from dodging their responsibilities by inappropriately relying on scientific imports (Yu, 2022). In this paper, my discussion of SR aims at adding substance to Philip Kitcher’s characterization of what a socially responsible scientist should do (Kitcher, 2001; Kourany, 2010). Socially responsible scientists should contribute their knowledge to good policies or should generally do science in order to enhance the social good, human flourishing, and so on. Socially beneficial consequences brought about by scientists exhibit their SR. I agree. In this section, I look into various processes of “making advisory claims” as a special form of action taken by the epistemic agents who were expected to carry out their non-epistemic missions. I show that the harmful consequences resulting from their advisory claims did not exhibit their SR, and argue that epistemic agents should be socially responsive by committing themselves to avoid making self-defeating claims.

Why do epistemic agents occasionally make self-defeating claims? For example, why do some claim to know something while they actually do not know? Why do some claim that there is nothing dangerous to come while in fact danger exists? They do not necessarily mean to bring about harmful consequences associated with such self-defeating claims with bad intentions. They might just tell white lies. Instead, I suggest that this is because the social roles individual epistemic agents play require them to make claims in certain ways, which might lead to wishful speaking (John, 2019). Moreover, it is not necessarily in itself a problem if individual epistemic agents play multiple social roles, for instance, scientists and officials. Problems arise, however, only when the multiple ways of communication involved can lead to their self-defeating, advisory claims, being counterproductive to their respective missions. According to Kitcher, such advisory claims exhibit their social irresponsibility. They failed their own goals because they made their advisory claims by dismissing other knowledge claims that could have otherwise enhanced their goals.

I suggest, aside from committing themselves to OEP, epistemic agents have an additional duty of keeping transparent the tensions between their knowledge and advisory claims associated with their non-epistemic missions. This is because SR demands epistemic agents to avoid making self-defeating claims. Such tensions might be irresolvable so that the epistemic agents have to, sometimes, distance themselves from certain non-epistemic missions, or just keep the epistemic and non-epistemic social roles they play separate.

For instance, the church authorities failed to offer a consistent epistemic account of why pious believers were punished by God’s wrath with earthquakes for no reason; the yatoi architects failed to explain why British architecture seemed vulnerable to earthquakes rather than robust, whereas they claimed to know how to avoid earthquake damages. They would have to modify their architectural theory based on a better understanding of the mechanics of earthquakes with higher accuracy, developed by instrument-based seismologists. In both cases, the advisory claims made by the church authorities and yatoi architects were self-defeating and resulted in underestimated harms on a short-term basis.

The latter two vignettes show that some epistemic agents could find it hard to live up to SR because they could not avoid making self-defeating claims. When the Chinese government and commissioned scientists actively blurred the distinction between earthquake specialists and lay observers for the sake of a shortage of trained personnel, laypeople could not advance the scientific tasks. They were not able to explain why not all macroscopic precursory phenomena indicated a large earthquake, and why not all large earthquakes had sufficiently observable precursory phenomena. However, this task had usually not been relegated to laypeople. When the government, commissioned scientists, and “mass scientists” mistakenly assumed the predictability of earthquakes, macroscopic precursory phenomena easily led to a cried wolf. In contrast, insufficient preparation and vigilance could happen when such phenomena did not appear. The socioeconomic costs of using these problematic estimations were not actually calculated. This was contradictory to the idea of their planned economy. Moreover, laypeople were supposedly not able to calculate them if this was possible at all. Worse, mixed quality of data collection made the mass science project more difficult to put into practice and to further develop it because the “mass scientists” were not adequately paid and not able to apply scientific methods rigorously. Forgetting to record, missing data, and filling out missing data from memory or resorting to guesswork were quite common. Verifying the mixed quality data from “mass scientists” by specialists was an additional large cost.

This blurring of the responsibilities of experts and non-experts demanded by the government complicated the task of earthquake prediction and conflicted with the governmental goal with which the policy of turning everyone to be a “mass scientist” was employed. Although the mass seismology project in China could be meaningful in terms of science education or science communication, which was of course socially valuable, the division between experts and non-experts for useful advice seems to remain practically necessary, for we saw that non-experts could complicate the implementation of national policies or even nullify the national goal. The social roles of lay observer and of scientist should thus be kept apart.

In contrast, the L’Aquila case exemplifies a common case of the social role that scientists or experts are appointed to be government officials, which is not alien to contemporary politics. They are expected to serve various governmental missions. We can furthermore see cases in intergovernmental organizations such as the WHO and the IPCC, in which scientists are appointed to make advisory claims to achieve intergovernmental missions, such as Tedros Adhanom Ghebreyesus in the WHO and Rajendra K. Pachauri in the IPCC.

Sometimes, the combined social role of scientist-official can become difficult to play because the value commitments of scientists and those of officials can be in tension, making advisory claims self-defeating. For example, what would it mean when a scientist-official claimed that “the swarms indicated no danger” or that “the swarms helped lessen the seismic potential by dissipating the accumulated seismic energy in this region?” Are they pure knowledge claims or advisory claims? For scientists have long known that roughly half of the large earthquakes had foreshocks, it is scientifically possible that a series of swarms indicate a coming large earthquake. Yet they also know that most swarms are just a series of small earthquakes that pose no serious danger (Hough, 2016). Thus, as scientists, the L’Aquila scientist-officials should have known that both scenarios are possible. They, however, chose to emphasize only one in their public announcements. This choice was partial in light of their full expertise.

Moreover, they actively excluded the warning of a coming earthquake from the heretical science. This exclusion led them to fail their political missions of civil protection. That said, their advisory claims came into conflict with their political missions, whereas their knowledge was compromised in the processes of communicating it, which could have been more comprehensive. The upshot is that making their advisory claims self-defeating, the scientist-officials neither fulfilled their role as scientists nor their role as officials.

Oreskes (2015) draws an interesting analogy of estimating this earthquake with climate change communication. She claims that earth scientists generally tend to underestimate risks, which has been criticized as mistakenly interpreting IPCC climate scientists’ model projections against actual observations (Pielke, 2019). However, her reason for translating this analogy to earthquake damage communication seems to be missing here for I find no basis for the claim that the scientist-officials either over-or underestimated the situation before the actual earthquake because they never aimed to “predict the unpredictable” (Hough, 2016). The L’Aquila scientists just said something that conflicted with their claimed epistemic aim. In Oreskes’ characterization of that episode, the scientist-officials actively avoided making a predictive claim and rejecting such a possibility since the beginning, whereas climate scientists generally make some projections. There is an apparent contrast between rejecting and accepting predictive claims.

My diagnosis of the problem with the scientists’ claim of no danger is that they made a self-defeating knowledge claim in conflict with what they knew. They, however, did not give such unreflective advice intentionally based on their knowledge but on wishful thinking, which resulted in wishful speaking. The problem with Oreskes’ evaluation of the scientists’ false reassurances is different (2005). She seems to implicate these scientists’ understatement in the scientists’ own acknowledgment of their inability to predict. A serious epistemic commitment to making no predictive claims, however, leads to neither overstatement nor understatement of future scenarios. Oreskes’ reappraisal of the L’Aquila case seems, thus, self-defeating if scientists make such a commitment as epistemic agents. I suppose this self-defeating reappraisal results from her hope to justify her claim that earth scientists tend to understate catastrophes, “so they should not.” If this is exactly her unstated normative assumption, then she might run the risk of falling into the problematic ideologically-driven research she argues against (Shaw, 2021). Her claim turns out to be unfounded and leads to a skew towards a particular error type. Either over-or understatement, in general, can be intertwined with specific ideologies and value judgments, while a predictive claim without either one is practically unavailable (see also Sect. 1.2). It is more important and realistic to make the hidden ideologies and value judgments more explicit if such predictive claims play a significant role in policymaking (which did not happen in the L’Aquila trial, Oreskes 2015), rather than giving groundless normative guidance to scientists.

Here, I propose two ways to avoid self-defeating advisory claims using the L’Aquila scientist-officials as my example.

The first solution is to split the social role of scientist-official. Scientists should make knowledge claims based on their expertise as comprehensive as possible; officials should make clear relevant socioeconomic considerations they have in mind. Their one-sided emphasis on the avoidance of overestimating socioeconomic damages from a future earthquake was unsatisfactory because such damages can be as expensive as underestimating damages. There should be no fundamental reason for preferring one over the other. As a result, the relatively comprehensive scientific expertise and socioeconomic considerations should be communicated to members of society, and let them decide what to do to best suit individual situations, and which risks are acceptable (Mulargia et al., 2017; Parker & Lusk, 2019). The scientists would thus avoid making self-defeating claims because they are not expected to carry out certain non-epistemic missions such as civil protection. Deciding which scenarios are dangerous to society is not part of their job. The officials, instead, have to take up the tasks of making judgments about scenarios deliberated by scientists and see whether they are dangerous (Betz, 2013).

The second solution is stressed if the first solution is not possible, which means that the officials should be scientists anyway. The scientists can still avoid making their advisory claims self-defeating by keeping their claims transparent as pure knowledge claims or as advisory claims. For individual scientists, that means they have an additional duty to keep their epistemic and non-epistemic goals separate to hold their made claims accountable. Their claims can be furthermore evaluated on epistemic and non-epistemic values involved, respectively. For instance, if they unavoidably had to claim “no danger” as a pure knowledge claim, they should add some complementary information such as “no danger means that in most cases small earthquakes are not followed by a large earthquake although we know that a large portion of large earthquakes has some small foreshocks.” As an advisory claim, they should make clear that “no danger means that we suggest you to just stay at home because we don’t want to scare you unnecessarily and bring about some unnecessary socioeconomic cost but it’s, of course, possible that we have unfortunately underestimated the risk, and you’d better stay in an open ground for two months avoiding such a potentially catastrophic earthquake.”

A similar struggle arose as many Western countries were reluctant to suggest the simple measure of face-masking in public in the early phase of the COVID-19 pandemic (Chen et al., 2021). When emphasizing that there is no scientific evidence regarding the efficacy of face-masking to avoid infection, did Dr. Tedros Adhanom Ghebreyesus in the WHO suggest “not to wear a face mask” because he made a pure knowledge claim as a scientist considering the relevant scientific grounds? Or rather, did he make an advisory claim as an international official careful not to expose the shortage of the global face mask supply? Did face-masking exactly require robust evidence with high scientific rigor, against the fact that most medical doctors have used them for a long time without scientifically robust evidence (Zagury-Orly, 2020; Howard et al., 2021)? The two ambivalent roles he played led him to give self-defeating advisory claims that delayed urgent, necessary action.

These two examples show that, sometimes, it could be an overloaded task if these epistemic agents were expected to carry out various non-epistemic missions. I suggest that, if overloaded, they should either split their social roles or keep their epistemic and non-epistemic goals transparent in order to keep the consequences resulting from their claims in check.

In this section, I attempt to add substance to Kitcher’s characterization of what a socially responsible scientist should do by clarifying what the historical epistemic agents should have done. His chief argument assumes that scientific knowledge can be translated into policymaking and that society should listen to scientists for advancing the social good and human flourishing (2001). I agree and would like to highlight some provisos. I argue that this translation without considering their epistemic and practical risks can lead to more harm than good. Moreover, whether it is always good to listen to or trust scientists should be extensively (re)evaluated on consequences brought about by scientists’ claims (Oreskes & Macedo, 2019). The deaths and the injured in the ruins after the L’Aquila earthquakes could have well listened to and trusted the scientists. Thus, socially responsible epistemic agents should actively engage in communicating with society about situations in which they find it difficult to share their knowledge *qua* epistemic agents AND to carry out their non-epistemic missions *qua* advisors or officials. They must tell society the harms they may bring about if they fail the expected roles they play.

## Concluding remarks

In this paper, I use four vignettes to contextualize three main channels where knowledge claims can result in conflicting advice due to epistemic and non-epistemic value commitments in tension. I stress that the value of openness to epistemic plurality and the value of social responsiveness should be constitutive of proper ethics of science communication for making advisory claims in public. Epistemic agents should commit themselves to these two values to avoid communication-induced risks. I argue that the value of openness to epistemic plurality is best suited to reduce the risks of prematurely or mistakenly excluding epistemic alternatives and possible courses of action, which are beneficial for science and society. This “avoidance of exclusion” has already had a harm-reducing effect on their mistaken claims. This value is, furthermore, conducive to alternative plurality and additive plurality in science. On the community level, the former may minimize its short-termed epistemic and practical risks and the latter may increase its long-termed reliability and credibility. Moreover, I sympathize with Kitcher’s account of socially responsible science and argue that we should include the commitment to avoid making self-defeating advisory claims and harmful wishful speaking. This is achieved by minimizing the values in tension embedded in the social roles individual epistemic agents play.

## Data Availability

Data sharing is not applicable to this article as no datasets were generated or analyzed during the current study.

## Footnotes

1 This structure was designed on a species of wasan (和算, Japanese mathematics) and Shintoism.

2 The reader may, however, doubt that the justification for my proposal, which relies on various counterfactual statements, is merely speculative and defeasible because advice based on the results of the counterfactual analysis may not always hold in complicated situations. Many factors other than such values could have made a difference in such an analysis. Values are, after all, not necessary like causes. I agree and think this doubt is constructive for approaching acceptable and desirable ethics of science communication. My proposal may work as a starter for this and should be examined extensively. In this regard, I suppose my proposal should be used to critically reflect on itself. In an attempt to show the inappropriateness of my proposal, my opponents might show that the commitment to the two suggested values does create more epistemic and practical risks resulting from speech acts, at least, in some cases. One might start with a statement like “if OEP and SR were not present in such and such cases, the situations in these cases could have been better.” Using a similar argument, John (2018), for instance, indicates the inappropriateness of values, such as openness and honesty, in some cases. If such cases were identified, my proposal would be shown to conflict with my set goal, so it fails to exhibit my social responsiveness (see also Sect. 4). Before such empirical scrutiny, I am not only open to alternative normative suggestions or alternative ethics of science communication (see also Sect. 3) that can better promote my goal but also curious about alternative justifications using no counterfactual statements. Note that such attempts are mostly based on a speculative assumption that some proper ethics of science communication are worth pursuing.
